# 

**Published:** 1859-03

**Authors:** 


NORTH AMERICAN

### MEDICO-CHIRURGICAL REVIEW.

Vol. III.] MARCH, 1859. [No. 2.

### CONTENTS.

### ANALYTICAL AND CRITICAL REVIEWS.

ART. PAGE

I.—1. On the Constitutional and Local Effects of Disease of the Supra-renal Capsules. By THOMAS ADDISON, M.D. . . . 193

2. Series of Cases Illustrating the Connection between Bronzed Skin and Disease of the Supra-renal Capsules. By JONATHAN HUTCHINSON, M.D. . . . . . . . . . 193

3. Recherches Experimentales sur la Physiologie et la Pathologie des Capsules Surrenales. Par le Docteur E. BROWN-SÉQUARD 193

4. Nouvelles Recherches Experimentales sur l'Importance des Fonctions des Capsules Surrenales. Par le Même. . . . 193

5. An Experimental Inquiry into the Functions of the Supra-renal Capsules, and their supposed Connection with Bronzed Skin. By GEORGE HARLEY, M.D. . . . . . . . . . . 193

6. The Sunburnt Appearance of the Skin as an Early Diagnostic Symptom of Supra-renal Capsule Disease. By ISAAC E. TAYLOR, M.D. . . . . . . . . . . . 193

II.—Lectures on the Diseases of Women. By CHARLES WEST, M.D. 216

III.—The Successful Treatment of Scarlet Fever; also, Observations on the Pathology and Treatment of Crowing Inspiration in Infants. By P. HOOD, Surgeon. . . . . . . . . 230

IV.—A Treatise on Rheumatic Gout, or Chronic Rheumatic Arthritis of all the Joints. By ROBERT ADAMS, M.D. . . . 239

V.—Catalogue of the Surgical and Pathological Museum of VALENTINE MOTT, M.D. . . . . . . . . . . . . 250

VI.—Anatomy, Descriptive and Surgical. By HENRY GRAY, F.R.S . 254

VII.—Contributions to Operative Surgery and Surgical Pathology. By J. M. CARNOCHAN, M.D. . . . . . . . . . 256

VIII.—First Principles of Physics or Natural Philosophy. By BENJAMIN SILLIMAN, JR., M.D. . . . . . . . . . 258

IX.—Selections from Favorite Prescriptions of Living American Practitioners. By HORACE GREEN, M.D. . . . . . 259

X.—A Treatise on Venereal Disease. By JOHN HUNTER, F.R.S. . 260

### ORIGINAL COMMUNICATIONS.

I.—Contributions to Obstetric Jurisprudence. (No. 2.) By HORATIO R. STORER, M.D. . . . . . . . . . . . . 260

II.—Medicine in China. By JOHN J. KERR, M.D. . . . . 282

III.—Exsection of the Sciatic Nerve for Neuralgia of the Stump following Amputation of the Thigh. By G. C. BLACKMAN, M.D. 298

IV.—Proceedings of the Pathological Society of Philadelphia. . 299

### AMERICAN PROGRESS OF MEDICAL SCIENCE.

Report on the Progress of Pathological Anatomy. By J. DA COSTA, M.D. 322

### BI-MONTHLY FOREIGN ABSTRACTS.

### PHYSIOLOGY AND CHEMISTRY.

ART. PAGE

I.—On the Suspension of the Radial Pulse in Forced Extension of the Arm . . . . . . . . . . . . 352
II.—Some Experimental Researches on the Effects of Cold on the Human Body . . . . . . . . . . 352
III.—On the Formation of Vivianite in the Animal Organism . . . 353

### PATHOLOGY.

IV.—On Apoplexy of the Cerebellum . . . . . . . . 353
V.—Observations on Pneumonia . . . . . . . . 355
VI.—On Rheumatism of the Diaphragm . . . . . . . . 356
VII.—On the Non-Elimination of Odorous Substances by the Urine in Bright's Disease . . . . . . . . . . 356
VIII.—A case of Spontaneous Hydrophobia . . . . . . . 357

### OBSTETRICS, AND DISEASES OF WOMEN AND CHILDREN.

IX.—Death after an Injection of Carbonic Acid into the Uterus . . . 358
X.—On the Treatment of Syphilis in Pregnant Women . . . . 359
XI.—Tubage of the Glottis by Forced Dilatation of the Larynx, in the Treatment of Croup . . . . . . . . . 360

### SURGERY.

XII.—On Two New Methods of Treating Diseases of the Lachrymal Sac. 361
XIII.—On the Use of Ergot in Certain Diseases of the Eye . . . . 362
XIV.—Observations on the Causes, Nature, and Treatment of Hemeralopia. 363
XV.—On the Internal Treatment of Ulcers of the Leg by Iodide of Potassium . . . . . . . . . . 363
XVI.—Modification in the preparation of Moxas . . . . . . 364

### BI-MONTHLY PERISCOPE.

### SURGERY.

1. Epilepsy for Thirty-two Years in a Man aged Forty-four; Operation of Castration . . . . . . . . . . . 365
2. Observations on the Treatment of some of the Symptoms of Syphilis. 367
3. On the Treatment of Hernia by Electricity . . . . . . . 369

### THERAPEUTICS.

1. Ligature of the Extremities in Intermittent Fever . . . . 370

EDITORS' TABLE.—Edibles in Negroland . . . . . . . . 372
Progress of American Surgery in Europe . . . . . . . 377
Pennsylvania Hospital.—Medical Students in the United States.—Medical Students Abroad.—Appointments in Medical Schools.—New Medical Journals . . . . . . 378
The Medical and Surgical Reporter.—American Medical Association.—Dr. Mütter's Donation.—Dr. Thomas Watson.—Sir Benjamin Brodie.—Prizes at the Académie de Médecine . . . . . 379
New Appointments at the Faculty of Medicine of Paris.—La Charité.—L'Hôtel-Dieu of Paris . . . . . . . . . . 380

NECROLOGICAL NOTICES.—Professor P. C. Gaillard, M.D. . . . 381
Dr. Richard Bright . . . . . . . . . . 382
Dr. J. C. W. Lever . . . . . . . . . . 383
M. Bonnet . . . . . . . . . . . 383
M. Bérard . . . . . . . . . . . 383

BI-MONTHLY BIBLIOGRAPHICAL RECORD . . . . . . . . 384

